# Automatic Quantification of Interstitial Lung Disease From Chest Computed Tomography in Systemic Sclerosis

**DOI:** 10.3389/fmed.2020.577739

**Published:** 2020-09-25

**Authors:** Alysson Roncally S. Carvalho, Alan R. Guimarães, Flávio R. Sztajnbok, Rosana Souza Rodrigues, Bruno Rangel Antunes Silva, Agnaldo José Lopes, Walter Araujo Zin, Isabel Almeida, Manuela Maria França

**Affiliations:** ^1^Department of Radiology, Medical School, Centro Hospitalar Universitário do Porto (CHUP), Instituto de Ciências Biomédicas Abel Salazar (ICBAS), Porto University, Porto, Portugal; ^2^Laboratory of Pulmonary Engineering, Biomedical Engineering Program, Alberto Luiz Coimbra Institute of Post-Graduation and Research in Engineering, Universidade Federal do Rio de Janeiro, Rio de Janeiro, Brazil; ^3^Laboratory of Respiration Physiology, Carlos Chagas Filho Institute of Biophysics, Universidade Federal do Rio de Janeiro, Rio de Janeiro, Brazil; ^4^Division of Pediatric Rheumatology, State University of Rio de Janeiro, Rio de Janeiro, Brazil; ^5^Department of Radiology, Universidade Federal do Rio de Janeiro, Rio de Janeiro, Brazil; ^6^IDOR - D'Or Institute for Research and Education, Rio de Janeiro, Brazil; ^7^Graduate Program in Medical Sciences, School of Medical Sciences, State University of Rio de Janeiro, Rio de Janeiro, Brazil; ^8^Clinical Immunology Unit, Deptartment of Medicine, Centro Hospitalar Universitário do Porto (CHUP), Instituto de Ciências Biomédicas Abel Salazar (ICBAS), Porto University, Porto, Portugal; ^9^Radiology Department, Centro Hospitalar Universitário do Porto (CHUP), Instituto de Ciências Biomédicas Abel Salazar (ICBAS), Porto University, Porto, Portugal

**Keywords:** systemic sclerosis, interstitial lung disease, chest computed tomography, quantitative chest CT-analysis, densitometry

## Abstract

**Background:** Interstitial lung disease (ILD) is a common complication in patients with systemic sclerosis (SSc), and its diagnosis contributes to early treatment decisions.

**Purposes:** To quantify ILD associated with SSc (SSc-ILD) from chest CT images using an automatic quantification method based on the computation of the weight of interstitial lung opacities.

**Methods:** Ninety-four patients with SSc underwent CT, forced vital capacity (FVC), and carbon monoxide diffusion capacity (DL_CO_) tests. Seventy-three healthy individuals without radiological evidence of lung disease served as controls. After lung and airway segmentation, the ratio between the weight of interstitial opacities [densities between −500 and +50 Hounsfield units (HU)] and the total lung weight (densities between −1,000 and +50 HU) was used as an ILD indicator (ILD[%] = 100 × [LW_(−500 to +50HU)_/LW_(−1, 000 to +50HU)_]). The cutoff of normality between controls and SSc was determined with a receiver operator characteristic curve. The severity of pulmonary involvement in SSc patients was also assessed by calculating *Z* scores of ILD relative to the average interstitial opacities in controls. Accordingly, SSc-ILD was classified as SSc Limited-ILD (*Z* score < 3) and SSc Extensive-ILD (*Z* score ≥ 3 or FVC < 70%).

**Results:** Seventy-eight (83%) SSc patients were classified as presenting SSc-ILD (optimal ILD threshold of 23.4%, 0.83 sensitivity, 0.92 specificity, and 0.94 area under the receiver operator characteristic curve, 95% CI from 0.89 to 0.96, 0.93 positive predictive value, and 0.81 negative predictive value, *p* < 0.001) and exhibited radiological attenuations compatible with interstitial pneumonia dispersed in the lung parenchyma. Thirty-six (38%) patients were classified as SSc Extensive-ILD (ILD threshold ≥ 29.6% equivalent to a Z score ≥ 3) and 42 (45%) as SSc Limited-ILD. Eighteen (50%) patients with SSc Extensive-ILD presented FVC < 70%, being only five patients classified exclusively based on FVC. SSc Extensive-ILD also presented lower DL_CO_ (57.9 ± 17.9% vs. 73.7 ± 19.8%; *p* < 0.001) and total lung volume (2,916 ± 674 vs. 4,286 ± 1,136, *p* < 0.001) compared with SSc Limited-ILD.

**Conclusion:** The proposed method seems to provide an alternative to identify and quantify the extension of ILD in patients with SSc, mitigating the subjectivity of semiquantitative analyzes based on visual scores.

## Key Results

- Quantitative analysis of chest CT scans by densitometry was able to quantify the extent of interstitial lung disease associated with systemic sclerosis (SSc).
- Extensive pulmonary involvement in SSc subjects was associated with pulmonary function impairment.
- Interstitial lung disease almost completely involves lung parenchyma in patients classified as with SSc extensive—interstitial lung disease.

## Introduction

Systemic sclerosis (SSc) is a rare multisystemic disease with heterogeneous clinical presentation characterized by extensive fibrosis and autoimmune and vascular dysfunction that may progress to multiple organ dysfunction including skin, lung, heart, and kidney involvement (1).

SSc clinical evolution is commonly insidious, progressive, and irreversible with considerable morbidity and mortality (2). The most common imaging and histopathologic pattern observed on computed tomography (CT) and surgical lung biopsy is nonspecific interstitial pneumonia (NSIP) (3, 4). On histopathology, this pattern is characterized by a relatively homogeneous thickening of the alveolar walls caused by inflammation (cellular-NSIP) and/or fibrosis (fibrotic-NSIP). In cellular-NSIP, alveolar septa are thickened by infiltration of lymphocytes and plasma cells, whereas in fibrotic-NSIP, the thickening is more related to collagen deposition (5). Chest CT findings usually reflect the histological pattern and are mainly characterized by ground-glass opacities, especially in cellular-NSIP. Interstitial reticular thickening with traction bronchiectasis and some focal consolidation are more frequently observed in fibrotic-NSIP (2).

As ILD associated with SSc (SSc-ILD) occurs in more than half of SSc patients (6) and as the extent of pulmonary involvement is quite variable, the severity of symptoms ranges from subclinical symptoms up to respiratory failure and death (7). Assuming that SSc-ILD considerably impacts morbidity and mortality, the early identification of patients with pulmonary involvement might help to mitigate both disease progression and severity of respiratory symptoms (2, 6).

CT is the gold standard for SSc-ILD detection (8). However, the visual quantification of ILD extension by CT generally results in low agreement even among experienced radiologists (9, 10). On the other hand, the quantitative assessment of chest CT, using dedicated software and methods, might overcome the observational limitation by quantifying the lung volume and tissue fraction in a voxel-by-voxel basis, allowing a more accurate calculation of the degree of pulmonary involvement (11, 12).

The main objective of this study was to assess whether the ratio between the weight of the interstitial opacities [densities between −500 and +50 Hounsfield units (HU)] and the total lung weight (LW) (densities between −1,000 and +50 HU) expressed in percentage values can be used as an ILD indicator. It is also intended to investigate the association between ILD severity and lung function impairment based on the assessment of forced vital capacity (FVC) and carbon monoxide diffusion capacity (DL_CO_).

## Materials and Methods

### Patients

This study reviewed CT and pulmonary function tests from 94 patients with SSc followed up at the Policlínica Piquet Carneiro da Universidade do Estado do Rio de Janeiro, Rio de Janeiro, Brazil, and at Centro Hospitalar Universitário do Porto, Porto, Portugal.

Patients had been diagnosed in accordance with ACR/EULAR criteria (13). Patients with clinical instability, history of respiratory infection 3 weeks before CT, who were previously or are currently smoking, with evidence of overlapping of scleroderma with other connective tissue diseases, and with a report of previous tracheal or pleuropulmonary disease not related to scleroderma were not included.

Cutaneous involvement was classified as limited (lc-SSc; thickening of the skin distal to the elbows and knees and proximal to the clavicles, including the face) or diffuse (dc-SSc; thickening of the proximal skin as well as of the skin distal to the elbows and knees and including the trunk and face) (14).

Anti-topoisomerase I (Scl-70) and anti-centromere (ACA) antibodies were determined by indirect immunofluorescence using Hep-2 cells as substrates, and autoantibody specificities were further assessed by ELISA (Shield, Dundee, UK).

Seventy-three none smoking healthy individuals with no chronic tracheal or pleuropulmonary diseases, chest CT scans without radiological abnormalities, and matched anthropometrically with SSc patients served as controls.

The protocol was approved at the Research Ethics Committee from the Universidade do Estado do Rio de Janeiro (CAAE-50752615.9.0000.5259) and Centro Hospitalar Universitário do Porto (reference number 2019.353, 288-DEFI/307-CE), and it complied with the current national and international standards.

### Pulmonary Function Tests

FVC, DL_CO_, and DL_CO_ adjusted to alveolar volume (DL_CO_/VA) were reviewed and expressed as percentages of the predicted values (15–18). Additionally, FVC(%)/DL_CO_(%) ratio was calculated in subjects with severe reduced DL_CO_ (<55% predicted) to assess isolated pulmonary hypertension (19). The maximal accepted interval between pulmonary function and CT acquisition was 3 months in SSc patients.

### Chest Computed Tomography Acquisition

CT scans were performed on a 64-channel multi-slice (Brilliance 40 scanner, Philips Medical Systems, Cleveland, OH, USA, and General Electrics Lightspeed VCT, Chicago Illinois, USA). The acquisitions were gathered in the axial plane with patients in the supine position with 120 kV and 120–300 mA (these parameters varied according to the biotype of the patient), slice thickness of 2 mm with 50% superposition. After the acquisition, all images were reconstructed with a matrix of 512 × 512/728 × 728 voxels using standard reconstruction algorithms.

### Imaging Processing

Lung segmentation and airway segmentation were performed (20), and images were then exported to an in-house developed software (QUALI) written in MATLAB® (MathWorks®, Natick, MA, USA).

Before densitometry calculation, the average densities expressed in HU of air inside the trachea (HU_Air_) and blood in the descending aorta (HU_Tissue_) were measured (QUALI software). The intensity values of all voxels in lung parenchyma were then linearly rescaled, considering that HU_Air_ and HU_Tissue_ should be equal to −1,000 and +50 HU, respectively (21, 22).

Total lung volume (TLV) was calculated as previously described (23). LW, in grams, was calculated as:

(1) $$\begin{array}{l} \text{LW }\left(\text{g}\right)=\left[\left(\text{HU}-{\text{HU}}_{\text{Air}}\right)/\left({\text{HU}}_{\text{Aorta}}-{\text{HU}}_{\text{Air}}\right)\right]\\ \times \text{voxel volume }\times \;1.04\text{ g}/\text{ml} \end{array}$$

where 1.04 mg/ml means lung tissue density, and HU is voxel density in an HU (21).

Additionally, LW related to hyperinflated (−1,000 to −901 HU), normally aerated (−900 to −501 HU), and to interstitial opacities (−500 to +50 HU) areas were also calculated and normalized to the whole LW (23).

### Regional Densitometry Assessment

LW was calculated in three sub-volumes equally divided considering the number of slices, from the basal to the apical (basal, middle, and apical lung thirds), and was used for regional densitometry assessment. LW at regions of interest generated by an erosion process performed from the outermost layer of the 10-mm-thick pulmonary parenchyma, progressing to the hilar regions in 10-mm steps, until the entire pulmonary parenchyma was also calculated.

### Identification of Systemic Sclerosis Patients With Interstitial Lung Disease

**Figure 1:**
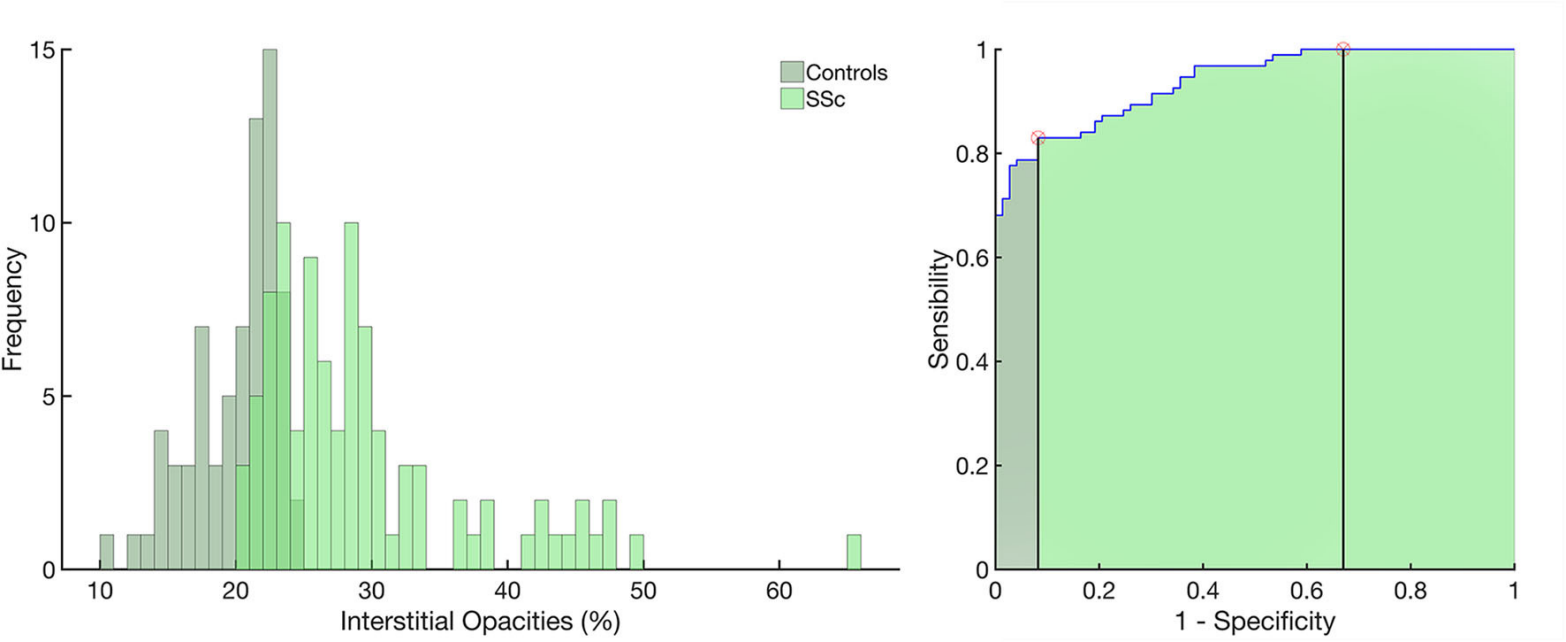
Left panel: histograms of the frequency of occurrence of interstitial opacities in the control group (light green) and in patients with systemic sclerosis (SSc, in dark green). Right panel: receiver operator characteristic curve with the area under the curve (AUC) hatched in light green. Vertical lines mark the normality cutoff (equivalent to 23.4% of total lung weight represented by interstitial opacites) and the *Z* score = 3 (equivalent to 29.6% of the total lung weight and used to classify SSc patients as with extensive interstitial lung disease, SSc-Extensive ILD). Between the normality cutoff up to *Z* score < 3, SSc patients were classified as with limited interstitial lung disease. Use of interstitial opacities as an indicator of ILD presented 0.83 sensitivity, 0.92 specificity, with an receiver operator characteristic curve with the area under the curve of 0.94; 95% from CI 0.89–0.96 (*p* < 0.001) and 0.93 positive predictive value and 0.81 negative predictive value.

After lung and airway segmentation, the ratio between the weight of voxels with densities between −500 and +50 HU and the total LW (100 × [LW_(−500 to +50HU)_/LW_(−1, 000 to +50HU)_]) was defined as an indicator of ILD. The cutoff of normality between controls and SSc was determined with a receiver operator characteristic (ROC) curve (Figure 1). The severity of pulmonary involvement in SSc patients was also assessed by calculating *Z* scores of ILD relative to the average interstitial opacities in controls. Accordingly, SSc-ILD patients were classified as SSc Limited-ILD (*Z* score < 3) and SSc Extensive-ILD (*Z* score ≥ 3). To increase sensitivity, all SSc patients with FVC < 70% were reclassified as SSc Extensive-ILD (24).

### Statistical Analysis

The normality of the data (Kolmogorov–Smirnov test with Lilliefors' correction) and the homogeneity of variances (Levene median test) were tested. As both conditions were always satisfied, all data are presented as mean and standard deviation.

From the ROC curve, the threshold limit, sensitivity, specificity, the area under the ROC curve, and bootstrapped 95% confidence intervals (CIs) (bias-corrected and accelerated), as well as positive and negative predictive values were assessed.

SSc-ILD and SSc without (SSc No-ILD) ILD were compared with a Student's *t*-test for independent samples. An ANOVA test followed by Bonferroni *post hoc* test assessed statistical differences among SSc Extensive-ILD, SSc Limited-ILD, and SSc No-ILD. Spearman correlation coefficient (r) was calculated to evaluate the associations between FVC, DL_CO_, and DL_CO_/VA with ILD-Extent in SSc patients. The criterion for determining significance was 5%. Statistical analysis was performed using Matlab® software (MathWorks®, Natick, MA, USA).

## Results

In SSc patients, the mean age at CT review was 54.5 ± 13.5 years, and 87 (92.5%) were women (*p* = 0.19 vs. controls). The disease durations were 4.21 ± 2.50 years from the onset of the non-Raynaud phenomenon and 9.65 ± 5.13 years from the onset of the Raynaud phenomenon. Sixty-four patients (68.1%) had lc-SSc, and 30 (31.9%) presented dc-SSc. Anti-topoisomerase I (Scl-70) antibody, anti-centromere antibody (ACA), and anti-RNA polymerase III were positive in 13 (13.8%), 50 (53.2%), and 3 (3.2%) patients, respectively. Twenty-eight patients (29.8%) did not present autoantibodies (Table 1).

**Table 1:** Demographic characteristics, clinical features, pulmonary function tests, and densitometry of the control group and scleroderma patients.

	**SSc (94)**	**Control group (73)**	***p*-value**
**Demographic data**			
Females	87 (92.5%)	64 (87.7%)	
Age (years)	54.5 **±** 13.5	57.9 **±** 20.0	0.19
BMI (kg/m^2^)	25.4 **±** 5.5	26.2 ± 4.3	0.27
**SSc cutaneous involvement**			
lc-SSc	64 (68.1%)	–	–
dc-SSc	30 (31.9%)	–	–
**Type of antibody**			
Anti-topoisomerase I antibody	13 (13.8%)	–	–
Anti-centromere antibody	50 (53.2%)	–	–
Anti-RNA polymerase III	3 (3.2%)	–	–
Autoantibody not identified	28 (29.8%)	–	–
**Lung function**			
FVC (% predicted)	98.0 **±** 26.9	–	**–**
DL_CO_/VA (% predicted)	81.3 **±** 18.6	–	**–**
DL_CO_ (% predicted)	69.9 **±** 20.8	–	**–**
**Densitometry**			
TLV (ml)	**3,804.8** **±** **1,160.9**	**4,170.7** **±** **755.3**	**0.021**
Total LT_Fraction_ (g)	**738.8** **±** **156.1**	**611.4** **±** **144.9**	**<0.001**
Hyperinflated (%)	**7.9** **±** **4.7**	**18.0** **±** **8.6**	**<0.001**
Normally aerated (%)	62.6 ± 7.1	62.0 ± 10.2	0.651
Interstitial opacities (%)	**29.5** **±** **8.1**	**20.0** **±** **3.2**	**<0.011**

*Values are presented as mean ± standard deviation or number (percentage value). Bold characters indicate statistically significant differences. BMI, body mass index; lc-SSc, limited skin form; dc-SSc, diffuse skin form; FVC (%), forced vital capacity expressed as predicted values; DL_CO_/VA, carbon monoxide diffusion capacity adjusted for alveolar volume expressed as predicted values; DL_CO_ (%), carbon monoxide diffusion capacity expressed as predicted values; TLV, total lung volume; Total LT_Fraction_, lung tissue fraction related to densities from −1,000 to +50 Hounsfield units (HU); Hyperinflated, tissue fraction related to densities from −1,000 to −901 HU; Normally Aerated, tissue fraction related to densities from −900 to −501 HU; Interstitial Opacities, lung weight related to densities from −500 to +50 HU*.

SSc presented a significant reduction in TLV, whereas total LW and LW related to hyperinflated and interstitial opacities were significantly higher in SSc compared with those in controls (Table 1).

Seventy-eight (83%) SSc patients were classified as presenting SSc-ILD based on the ROC curve with an optimal ILD threshold of 23.4%, 0.83 sensitivity, 0.92 specificity, and 0.94 area under the ROC curve; 95% from CI 0.89–0.96 (*p* < 0.001), 0.93 positive predictive value, and 0.81 negative predictive value (Figure 1).

**Figure 2:**
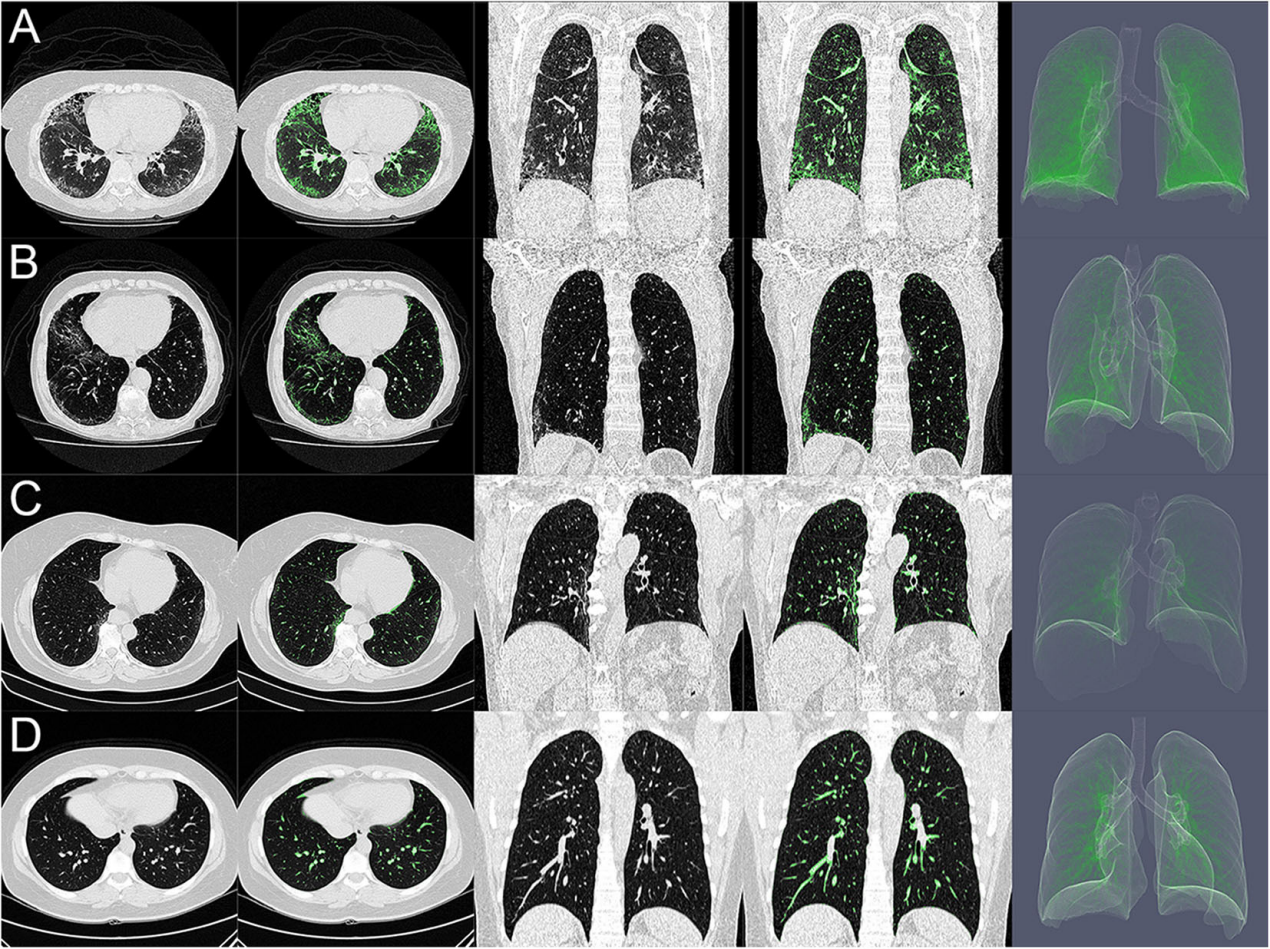
**(A–D)** Show axial (columns 1 and 2) and coronal (columns 3 and 4) CT scans and three-dimensional rendering (column 5) of the lung of systemic sclerosis classified as with extensive interstitial lung disease (SSc Extensive-ILD, **A**), as with limited interstitial lung disease (SSc Limited-ILD, **B**), as with no interstitial lung disease (SSc No-ILD, **C**), and controls **(D)**. Note, in **(A)**, predominance of ground glass/reticular consolidation and compressed tissue likely related to cellular/fibrotic nonspecific interstitial pneumonia at basal lung third. Those in green are represented interstitial opacities with densities ranging from −500 and +50 HU that would represent the extent of pulmonary involvement.

Thirty-six (38%) patients were classified as SSc Extensive-ILD (ILD threshold of 29.6%, 0.32 sensitivity, and 1.00 specificity) and exhibited radiological attenuations compatible with interstitial pneumonia diffusely distributed in the lung parenchyma (Figure 2, uppermost row). Forty-two (45%) patients were classified as SSc Limited-ILD, and radiological attenuations were mainly distributed in basal and middle lung thirds (Figure 2, second row). In SSc patients, SSc-ILDs, color-coded in green, were therefore interpreted, in addition to the peribronchial vessels, as ground-glass opacities, reticular consolidations, and possible compressed tissues broadly spread over the lung parenchyma (Figure 2, rightmost column). Note that the pattern of involvement of lung parenchyma differed between Extensive- and Limited-ILDs.

In controls, interstitial opacities mainly represented peribronchial vessels and some ground glass (Figure 2, lowermost row), especially in dorsal–basal-dependent regions in older subjects (age ≥ 60 years) being quite comparable with SSc No-ILD patients (Figure 2, third row).

Eighteen (50%) patients with SSc Extensive-ILD presented FVC < 70%, being five patients initially classified as SSc Limited-ILD, and in only three SSc (8%) Extensive-ILD, a severe restrictive pattern (FVC < 50%) was observed. Moreover, SSc Extensive-ILD presented lower DL_CO_ (57.9 ± 17.9% vs. 73.7 ± 19.8%; *p* < 0.001) and TLV (2,916 ± 674 vs. 4,286 ± 1,136, *p* < 0.001), hyperinflated, and normally aerated tissue fraction, but not lower DL_CO_/VA and total LW compared with those in SSc Limited-ILD (Table 2).

**Table 2:** Demographic variables, pulmonary function tests, and densitometry considering scleroderma patients with less or greater pulmonary involvement.

	**SSc-ILD (78)**	**SSc No-ILD (16)**	***p*-value**	
	**SSc extensive- ILD (36)**	**SSc limited-ILD (42)**		
**Demographic data**				
Females	34 (94%)	38 (90%)	15 (94%)	
Age (years)	51.7 ± 14.6	58.9 ± 12.3	49.5 ± 11.0	
BMI (kg/m^2^)	25.8 ± 5.9	25.0 ± 5.7	25.4 ± 4.4	
**SSc cutaneous involvement**				
lc-SSc	22 (23%)	30 (32%)	12 (13%)	
dc-SSc	14 (15%)	12 (13%)	4 (4%)	
**Type of antibody**				
Anti-topoisomerase I antibody	4 (4%)	5 (5%)	4 (4%)	
Anti-centromere antibody	23 (24%)	25 (27%)	2 (2%)	
Anti-RNA polymerase III	–	2 (2%)	1 (1%)	
Autoantibody not identified	9 (9%)	10 (11%)	9 (9%)	
**Pulmonary function tests**				
FVC (% predicted)	**74.6** **±** **22.0**^**a**^^,^ ^**b**^	**112.1** **±** **18.5**	**113.5** **±** **17.0**	**<0.001**^**a**^^,^ ^**b**^
50% < FVC < 70%	18 (50%)	0 (0%)	0 (0%)	
FVC < 50%	3 (8%)	0 (0%)	0 (0%)	
DL_CO_/VA (% predicted)	84.1 **±** 20.2	76.2 **±** 18.5	88.3 **±** 11.4	
DL_CO_ (% predicted)	**57.9** **±** **17.9**^**a**^^,^ ^**b**^	**73.7** **±** **19.8**^**c**^	**87.0** **±** **13.3**	**<0.001**^**a**^^,^ ^**b**^ **0.04**^**c**^
DL_CO_ <55%	17 (47%)	10 (24%)	0 (0%)	
55% < DL_CO_ <80%	14 (39%)	12 (29%)	5 (31%)	
FVC(%)/DL_CO_(%)	1.35 ± 0.38	1.62 ± 0.49	1.33 ± 0.25	**0.02**^**a**^
FVC(%)/DL_CO_(%) > 1.4 and DL_CO_(%) <55%	8 (22%) **1.86** **±** **0.28**^**a**^	9 (21%) **2.35** **±** **0.48**	0 (0%)	**0.022**
**CT densitometry**				
TLV (mL)	**2,915.7** **±** **673.9**^**a**^^,^ ^**b**^	**4,286.3** **±** **1,136.5**	**4,541.0** **±** **800.4**	**<0.001**^**a**^^,^ ^**b**^
Total lung weight (g)	723.3 **±** 142.1	736.1 **±** 158.7	780.7 **±** 180.9	
Hyperinflated (%)	**4.6** **±** **3.1**^**a**^^,^ ^**b**^	**9.8** **±** **4.3**	**10.2** **±** **4.5**	**<0.001**^**a**^^,^ ^**b**^
Normally aerated (%)	**58.7** **±** **8.4**^**a**^^,^ ^**b**^	**63.9** **±** **4.5**	**67.8** **±** **4.9**	**0.002**^**a**^ **<0.001**^**b**^
ILD Extent (%)	**36.7** **±** **8.7**^**a**^^,^ ^**b**^	**26.3** **±** **2.1**^**c**^	**22.0** **±** **0.8**	**<0.001**^**a**^^,^ ^**b**^ **0.03**^**c**^

*Values are presented as mean ± standard deviation. Bold characters indicate statistically significant differences*.

a *Statistically significant difference between SSc Extensive-ILD and SSc Limited-ILD*.

b *Statistically significant difference between SSc Extensive-ILD and SSc No-ILD*.

c *Statistically significant difference between SSc Limited-ILD and SSc No-ILD*.

*SSc No-ILD, scleroderma with no interstitial lung disease (ILD ≤ 25%); SSc-ILD, scleroderma with interstitial lung disease; SSc Limited-ILD, scleroderma with interstitial lung disease between 25 and 35%; SSc Extensive-ILD, scleroderma with interstitial lung disease higher than 35%; BMI, body mass index; FVC (%), forced vital capacity expressed as predicted values; DL_CO_/VA, carbon monoxide diffusion capacity adjusted for alveolar volume expressed as predicted values; DL_CO_ (%), carbon monoxide diffusion capacity expressed as predicted values; TLV, total lung volume; Total lung weight, lung tissue fraction related to densities from −1,000 to +50 Hounsfield units (HU); Hyperinflated, tissue fraction related to densities from −1,000 to −901 HU; Normally Aerated, tissue fraction related to densities from −900 to −501 HU; ILD Extent, extent of interstitial lung disease defined as lung weight related to interstitial opacities normalized by total lung weight*.

Moreover, the proportion of FVC/DL_CO_ ratio higher than 1.4 in subjects with severe DL_CO_ reduction (DL_CO_ < 55%) was very similar to that in patients with Extensive- and Limited-ILDs (22 and 21%, respectively).

Noteworthy, in five (31%) SSc No-ILD patients, a moderate reduction in DL_CO_ (55% < DL_CO_ < 80%) was observed with DL_CO_ ranging from 62 to 76% despite presenting no evidence of a restrictive pattern in spirometry. Additionally, the frequencies of occurrence of positive anti-topoisomerase I (ATA-1) and anti-centromere antibodies (ACA) were quite similar among SSc patients with no apparent relationship with ILD extent (Table 2).

**Figure 3:**
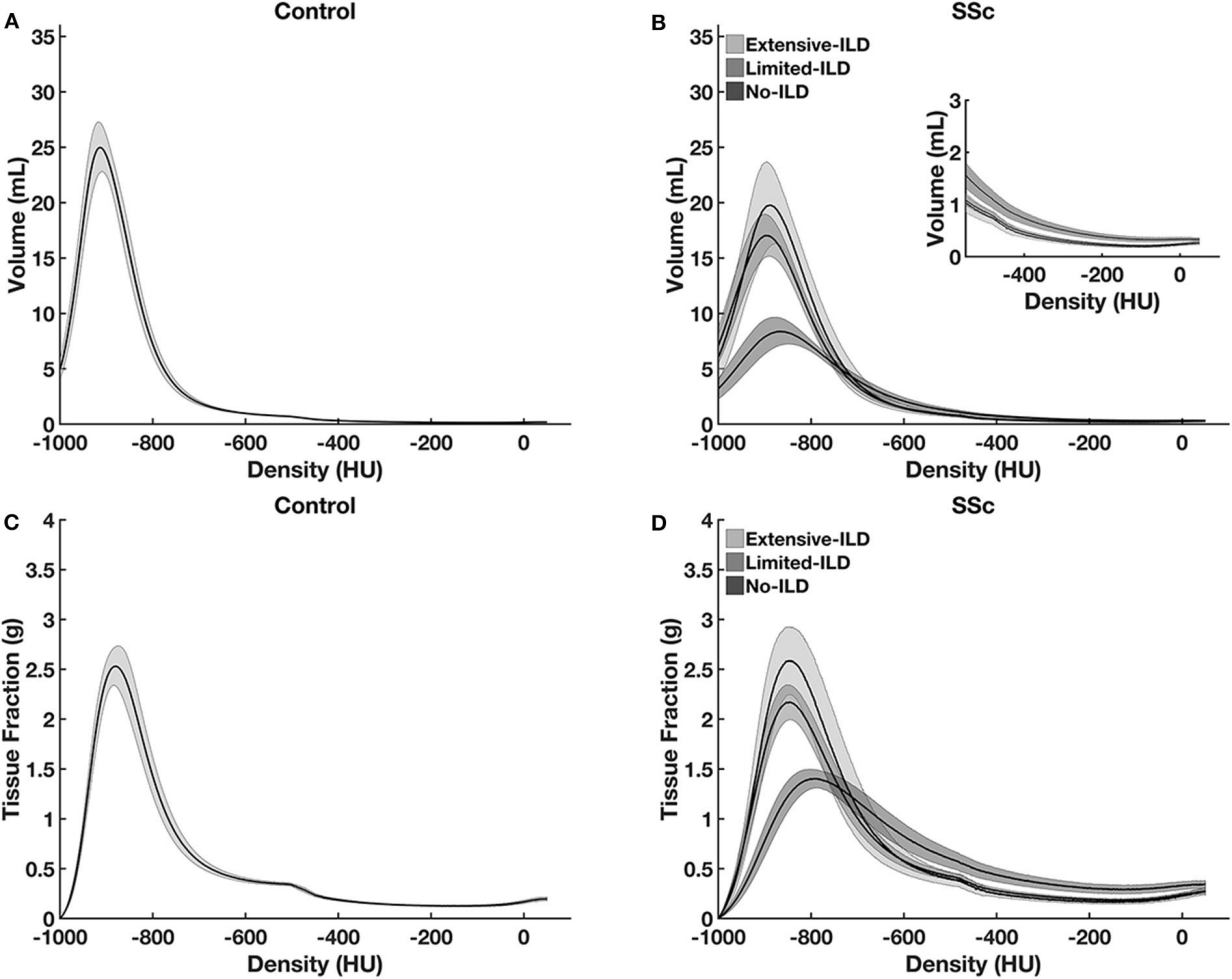
Lung tissue volume **(A,C)** and lung tissue fraction **(B,D)** plotted against density in controls **(A,B)** and SSc-patients **(C,D)**. Note the peak volume and tissue fraction reduction and the right-shift of the histogram to higher densities from SSc No-ILD to SSc Extensive-ILD. Such aspect reflects lung volume reduction as well as tissue increase at higher densities, as ILD spreads through lung parenchyma. Graphs are presented with average, and the hatched areas correspond to the 95% coefficient interval.

In Figure 3, volume and tissue fractions are plotted against densities in HU. A reduction in the peak of volume and tissue fractions at densities related to normally aerated areas and a right-shift toward densities related to ground-glass opacities and consolidation were observed. In the Supplementary Material, videos of representative cases of SSc Extensive-ILD, Limited-ILD, and No-ILD are presented in axial and coronal slices (Supplementary Videos 1–6). Thus, it is possible to compare how tissue fractions vs. density relationships of patients with SSc differ from the control group, as the lung is visualized in axial and coronal sections.

**Figure 4:**
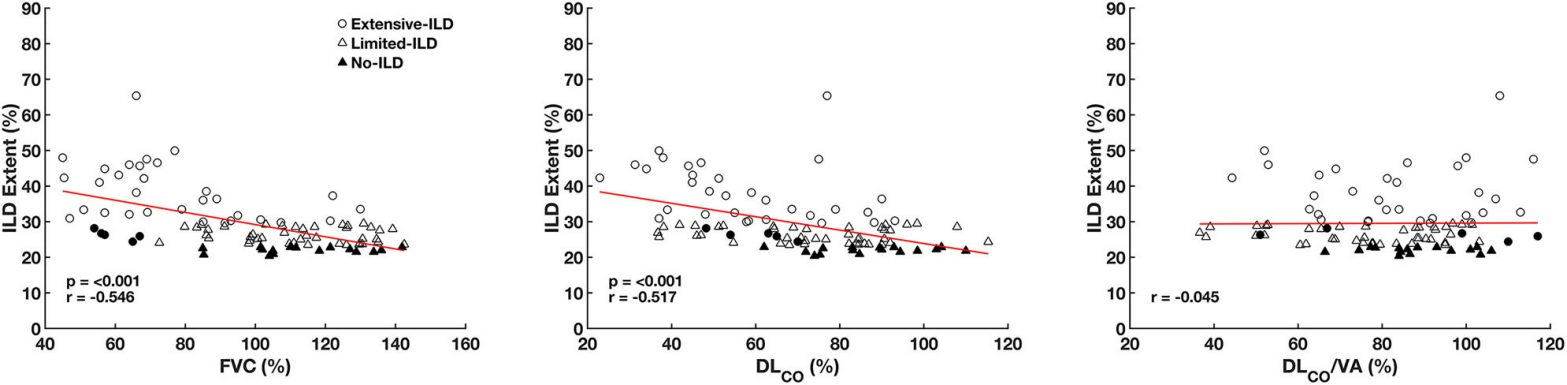
Correlation between interstitial lung disease extent (ILD Extent), forced vital capacity (FVC), carbon monoxide diffusion capacity (DL_CO_), and carbon monoxide diffusion capacity adjusted to alveolar volume (DL_CO_/VA) expressed as predicted values considering all SSc patients. Circle symbols represent SSc Extensive-ILD, SSc Limited-ILD triangles, and SSc No-ILD patient filled triangles. Filled circles represent patients initially classified as SSc Limited-ILD but with an FVC < 70% being reclassified as SSc Extensive-ILD.

Figure 4 presents the correlations between ILD extent, FVC (left panel), DL_CO_ (middle panel), and DL_CO_/VA (right panel). A significant inverse and moderate to poor correlation were observed between ILD Extent and FVC (*r* = −0.57, *p* < 0.001) and between ILD extent and DL_CO_ (*r* = −0.49, *p* < 0.001) but not with DL_CO_/VA (*r* = 0.009, *p* = 0.99).

**Figure 5:**
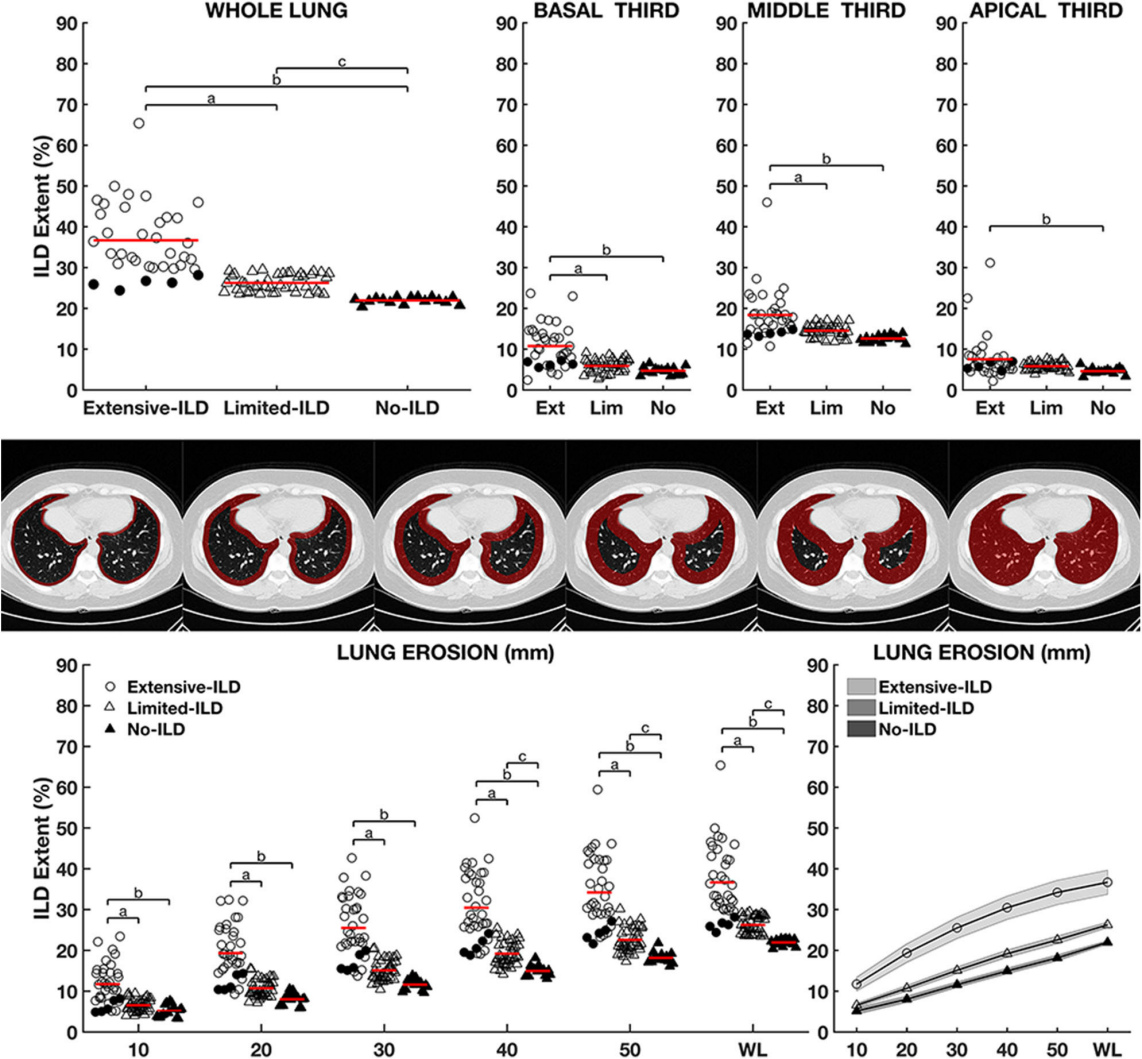
Upper panels: Interstitial lung disease extent (ILD Extent) expressed as a percentage of total lung weight among groups considering the whole lung, basal, middle, and apical thirds. Lower panels: comparison between groups at different erosion depths from the outermost layer of the parenchyma hilar regions, outlined in the middle panels. Left panel shows ILD Extent in 10-mm steps, and right panel shows the continuous graphs with mean and 95% coefficient interval. Letters a, b, and c represent statistically significant differences between SSc Limited-ILD vs. No-ILD, SSc Diffuse-ILD vs. No-ILD, and SSc Diffuse-ILD vs. SSc Limited-ILD, respectively. Circle symbols represent SSc Extensive-ILD, SSc Limited-ILD and SSc No-ILD triangles, and patient filled triangles. Filled circles represent patients initially classified as SSc Limited-ILD but with an FVC < 70% being reclassified as SSc Extensive-ILD. Noteworthy, ILD Extent in SSc Extensive-ILD group was always significantly higher than in SSc Limited- and SSc No-ILD even in the subpleural surface (Figure 4, lower panels).

Figure 5, upper panels, depicts ILD extent in the whole lung and the basal, middle, and apical thirds. Additionally, the extent of the pulmonary involvement from the subpleural layers to hilar regions could be observed in SSc No-ILD and SSc Limited- and Extensive-ILDs (Figure 5, lower panels).

## Discussion

The main findings of the present study were (1) an optimal threshold of 23.4% of LW assigned as interstitial opacities was determined by ROC curve and used to classify 78 SSc patients as presenting ILD; (2) 36 (38%) SSc patients were classified as SSc Extensive-ILD and 42 (45%) as SSc Limited-ILD based on the *Z* score for interstitial opacities in patients with SSc relative to controls; (3) ILD extent negatively and moderately to poorly correlated with FVC and DL_CO_, but not with DL_CO_/VA; and (4) in patients with SSc Extensive-ILD, pulmonary involvement dispersed in all lung parenchyma from apical to basal thirds and from the periphery to hilar regions.

In healthy subjects, CT interstitial opacities are mainly related to vascular peribronchial network and, especially in older subjects and at dorsal-dependent regions, to areas with low ventilation/perfusion ratio (25–28). Thus, we speculate that tissue weight related to such opacities could be used as a potential indicator of ILD in SSc patients. Hence, the laborious exclusion of peribronchial vessels would not be necessary, and values above a certain normality threshold could be interpreted as ILD in patients with SSc.

The ROC curve from the weight of interstitial opacities in controls and SSc patients determined the optimal limit of normality of 23.4% of tissue weight related to interstitial opacities (Figure 1). The visual inspection of CT matched with computed interstitial opacities indicated that, in addition to peribronchial vessels, ground-glass opacities and reticular/focal consolidations were also identified (Figure 2) and could represent more than 40% of the total LW in one-third of patients classified as SSc Extensive-ILD (Figures 1, 5).

The prevalence of ILD based on our proposed criteria was around 83% and is just slightly larger than previously reported data (prevalence of SSc-ILD up to 80%) (29). A spread distribution of ILD was observed in patients classified as SSc Extensive-ILD involving basal, middle, and apical lung thirds as well as lung periphery and central regions. On the other hand, SSc Limited-ILD pulmonary involvement concerned basal and middle thirds more extensively than SSc No-ILD (Figure 4).

An impaired pulmonary function was observed in patients classified as SSc Extensive-ILD with 58% presenting moderate/severe restrictive pattern in spirometry without airway obstruction and 86% presenting moderate/severe reduction in lung diffusion capacity. FVC is the main marker of restrictive ventilatory pattern, which is likely to be associated with fibrosis, whereas DL_CO_ is an indicator of alveolitis, ventilation–perfusion mismatch, vascular involvement, and fibrosis (27).

DL_CO_ seems to be more sensitive in detecting lung involvement than FVC, although it is less specific regarding ILD, as pulmonary vascular disease and coexisting fibrosis may also lead to decreased DL_CO_ (27, 28). Thus, a moderate and isolated reduction in DL_CO_ may also indicate just subclinical alveolitis (19), which might have occurred in 5 (31%) of our patients classified as SSc No-ILD.

Interestingly, no significant differences among SSc-ILD subjects were observed in terms of DL_CO_/VA (Table 2). DL_CO_/VA more specifically represents diffusion, as the transfer factor is clearly related to available lung surface estimated by VA (27). Thus, one could speculate that the observed DL_CO_ reduction in SSc Extensive-ILD was mainly associated with isolated pulmonary hypertension and not to a reduction of lung surface available for gas exchange.

However, the same proportion of SSc Extensive- (22%) and Limited-ILD patients (21%) had both DL_CO_ and FVC severely reduced, thus presenting an FVC/DL_CO_ ratio higher than 1.4, stressing that isolated pulmonary hypertension could not exclusively explain the reduction in DL_CO_ (19) both in Extensive- and Limited-ILDs.

The main limitation of this study is the small number of SSc patients. This aspect could have an impact on the power of our study. However, patients included herein were well characterized clinically, radiographically, and functionally. Another important limitation is the need for a threshold of normality for ILD determination in SSc patients as well as a severity classification, with *Z* score mainly related to the average weight of interstitial opacities in controls. Although an impaired pulmonary function was observed in SSc Extensive-ILD, it is not yet possible to assess whether those classified as SSc Limited-ILD would benefit from any specific antifibrotic therapy (30).

Finally, the application of densitometry is not the only method for quantitative evaluation in chest CT images. Other methods based on deep-learning or even texture analysis (31, 32) have been successfully applied to similar subjects. However, densitometric assessment methods are computationally very simple and do not require prior preparation of libraries or image banks with their respective radiological pattern classification.

In conclusion, SSc-ILD could be automatically quantified by computing LW related to interstitial opacities in densities ranging from −500 to +50 HU. Patients with SSc, who had an ILD extent greater than about 30% of total LW, exhibited moderate to severe restrictive pattern and reduced diffusion capacity with ILD spread throughout the entire lung parenchyma. The proposed method seems to provide an alternative to estimate ILD in patients with SSc, mitigating the subjectivity of semiquantitative analyzes based on visual scores. Further follow-up studies of SSc patients using the presently described method could clarify the extent of pulmonary involvement and suggest the therapeutic window for pulmonary fibrosis-inhibiting drugs.

## Data Availability Statement

All datasets presented in this study are included in the article/Supplementary Material.

## Ethics Statement

The protocol was approved at the Research Ethics Committee from the UERJ (CAAE-50752615.9.0000.5259) and from CHUP (reference number 2019.353, 288-DEFI/307-CE), and it complied with the current national and international standards. The ethics committee waived the requirement of written informed consent for participation. Written informed consent was obtained from the individual(s) for the publication of any potentially identifiable images or data included in this article.

## Author Contributions

AC: image processing and analysis of results, statistical evaluation, theoretical development of software, the computation method of voxel-to-voxel analysis, and writing of the text and submission of the article. AG: image processing and segmentation and statistical evaluation. FS and MF: capture and organization of clinical data and draft review. RR: capture and organization of clinical data, discussion of results, and draft review. BS, AL, and IA: capture and organization of clinical data. WZ: draft review. All authors contributed to the article and approved the submitted version.

## Conflict of Interest

The authors declare that the research was conducted in the absence of any commercial or financial relationships that could be construed as a potential conflict of interest.

## Supplementary Material

Supplementary Videos Axial (Videos 1–3) and coronal (Videos 4–6) chest CT scans of representative patient with systemic sclerosis classified as with extensive (SSc Extensive-ILD, Video 1), limited (SSc Limited-ILD, Video 2) and with no interstitial lung disease (SSc No-ILD, Video 3). Lung tissue fraction is plotted against density in controls, as mean (white line) and one standard deviation (hatched area). In each slice the plot of cumulative lung tissue fraction against density is presented in each SSc subgroups (green line). Note the progressive separation from the control group. For comparison purposes, the average values of hyperinflated areas, normally aerated and the tissue weight related to interstitial opacities are presented (outermost right panels) in the control group (gray) and in each group of patients with systemic sclerosis (red bars).
